# Knowledge domain and emerging trends in beta‐cell research: A bibliometric and knowledge‐map analysis

**DOI:** 10.3389/fendo.2022.1086667

**Published:** 2023-01-19

**Authors:** Yunpeng Luo, Tong Wang, Zhuhong Chen, Guangde Zhang

**Affiliations:** ^1^ Graduate School, Beijing University of Chinese Medicine, Beijing, China; ^2^ Institute of Endocrinology, Xiyuan Hospital, China Academy of Chinese Medical Sciences, Beijing, China

**Keywords:** bibliometric, knowledge-map, Citespace, VOSviewer, insulin-secreting cells

## Abstract

**Background:**

Up to now, the physiology, pathology, and recovery of beta-cells have been intensively studied and made great progress, and these are of major significance for the treatment of related diseases. Nevertheless, a comprehensive and objective report on the status of beta-cell research is lacking. Therefore, this study aims to conduct a bibliometric analysis to quantify and identify the current status and trending issues in beta-cell research.

**Methods:**

The articles and reviews related to beta-cell were obtained from the Web of Science Core Collection on August 31, 2022. Two scientometric software (CiteSpace 6.1.R3 and VOSviewer 1.6.18) were used to perform bibliometric and knowledge-map analysis.

**Results:**

A total of 4098 papers were published in 810 academic journals in 2938 institutions from 83 countries/regions. The number of beta-cell-related publications was increasing steadily. The United States was the most productive country, while Universite libre de Bruxelles, University of Toronto and University of Geneva were the most active institutions. *Diabetes* published the most beta-cell studies and received the largest number of co-citations. Decio I Eizirik published the most papers and had the most co-citations. Twelve references on reviews and mechanisms were regarded as the knowledge base. Four major aspects of beta-cell research included the pathological mechanism of beta-cell failure, the recovery of beta cells, the risk factor related to beta cells, and the physiology of beta cells. Endoplasmic reticulum stress and oxidative stress have been core elements throughout the research in this field. In addition, beta-cell dedifferentiation, inflammation, autophagy, miRNA, and lncRNA are hot topics nowadays. Additionally, stem cell replacement therapies might be the alternative way to reverse beta-cell failure. Restoring beta-cell mass and function will remain a research goal in the future.

**Conclusion:**

This study provided a comprehensive overview of beta-cell research through bibliometric and visual methods. The information would provide helpful references for scholars focusing on beta cells.

## 1 Introduction

Insulin-secreting cells, also known as pancreatic beta cells, are the main component of pancreatic islets. Insulin is stored in crystalline form as a Zn_2_-insulin_6_ complex in secretory vesicles, and accounts for 5-10% of the total protein content of beta cells (1). It is released by regulated cytosolic emesis. Insulin secretion is regulated by several factors, including glucose concentration, most amino acids, fatty acids (2), hormones, neurotransmitters, etc. Alterations in insulin secretion are associated with various disorders, such as diabetes, hypoglycemic states, and cardiometabolic diseases (3). Up to now, the physiology, pathology, and recovery of beta-cells have been intensively studied, and made great progress, and these are of major significance for the treatment of related diseases. However, there is no comprehensive and objective report on publishing trends, influential authors or institutions and their collaborations, the knowledge base, the evolution of hotspots, or the emerging topics in beta-cell research to our knowledge.

This study was designed to objectively describe the knowledge base and emerging trends of beta-cell research using two commonly used bibliometric tools, CiteSpace and VOSviewer. Firstly, we aimed to quantify and identify the general information in beta-cell research, such as the individual impact and the cooperation information, by analyzing annual publications, countries/regions, institutions, authors and co-cited authors, journals and co-cited journals. Secondly, we evaluated the knowledge base on beta-cell research using an analysis of co-cited references. Finally, keyword analysis and co-cited reference burst analysis were used to find the knowledge structure and hotspots evolution and detect the emerging topics of beta-cell research.

## 2 Materials and methods

### 2.1 Data collection

The Web of Science Core Collection (WoSCC) database is commonly used for bibliometric analysis. It can provide comprehensive information and is regarded as the most influential database (4, 5). Therefore, we chose it as the data source for our study.

Literature was extracted from the WoSCC database and downloaded on August 31, 2022. In order to ensure the highest accuracy and minimum acceptable error, we chose the title search (6). The search terms were as follows: [TI= (“Insulin Secreting Cells” or “Insulin-Secreting Cells” or “Insulin-Secreting Cell” or “Pancreatic B Cells” or “Pancreatic B Cell” or “Pancreatic beta Cells” or “Pancreatic beta Cell”)] AND [Publication type = (Article or review)] AND [Language = (English)]; Publication data: “1985-01-01” to “2022-08-31”. Search results were downloaded as “Full Record and Cited References” and “Plain Text”. For further analysis, we subsequently renamed the files as “download_*.txt”, which CiteSpace software could read (5).

### 2.2 Data analysis

We used CiteSpace 6.1.R3, VOSviewer 1.6.18, and Microsoft Excel 2021 to perform bibliometric analysis and visualization. We cleaned the data before analyzing, for instance, “pancreatic beta-cells”, “pancreatic beta-cell”, “pancreatic beta cells”, “beta cell”, “beta-cell”, “pancreatic beta cell”, “beta cells”, and “beta-cells” were merged as “insulin-secreting cells”; “endoplasmic reticulum stress” and “ER stress” were unified as “ER stress”.

CiteSpace is a bibliometric and visual analysis tool that specializes in detecting collaborations, internal structures, key points, potential trends, and dynamics in a scientific field (7). Therefore, we used CiteSpace to analyze the co-cited references, reference bursts, and keyword bursts. The settings were as follows: timespan (2012–2022), years per slice (1), pruning (none), selection criteria (Top N=100), minimum duration of burstness (2 years), and others followed the default (5).

VOSviewer is another bibliometric software adept at creating and visualizing knowledge maps, showing the types of clusters, overlays, or density colors (8). It was used to perform the co-occurrence of Countries/Regions and Institutions, authors and co-cited authors, journals and co-cited journals, and keywords. We set the counting method as fractional counting (4), other thresholds were shown in the corresponding chapter.

We used Microsoft Office Excel 2021 to analyze the trend of the annual publications. Moreover, the impact factor (IF) and Journal citation reports (JCR) division of journals and the H-index of scholars were obtained from the Web of Science on September 7, 2022.

## 3 Results

### 3.1 The trend of publication outputs

**Figure 1:**
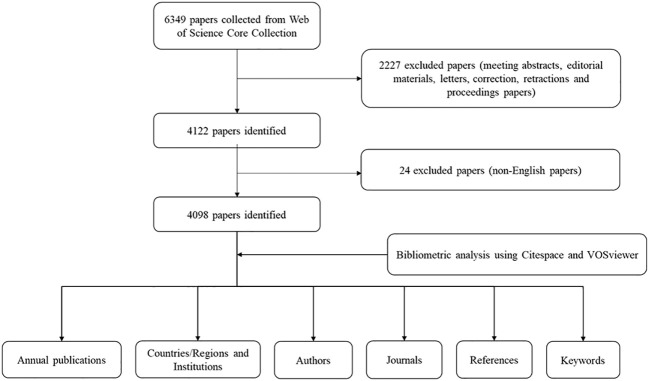
Flow chart of the data collection for research on beta-cell research.

**Figure 2:**
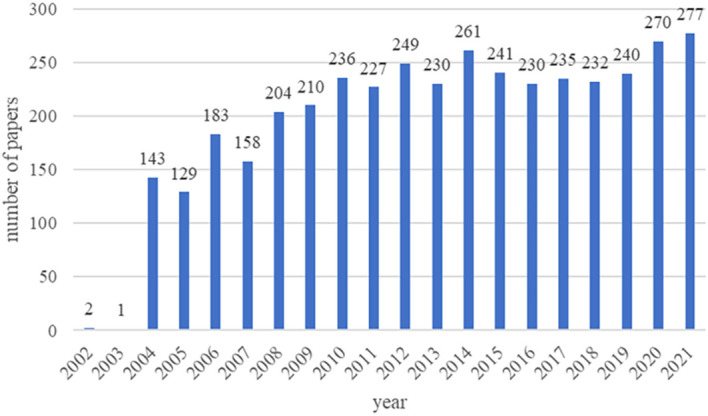
Trends of beta-cell publications over the past 20 years.

A total of 6349 papers were retrieved. Among them, 2227 unqualified articles including meeting abstracts, editorial materials, corrections, letters, retractions, proceedings paper, and 24 non-English papers were excluded. Finally, a total of 4098 eligible papers were included (**Figure 1**). As shown in **Figure 2**, the number of papers was low from 2002 to 2003 and has been increasing steadily from 2004 to now.

### 3.2 Distribution of countries/regions and institutions

**Figure 3:**
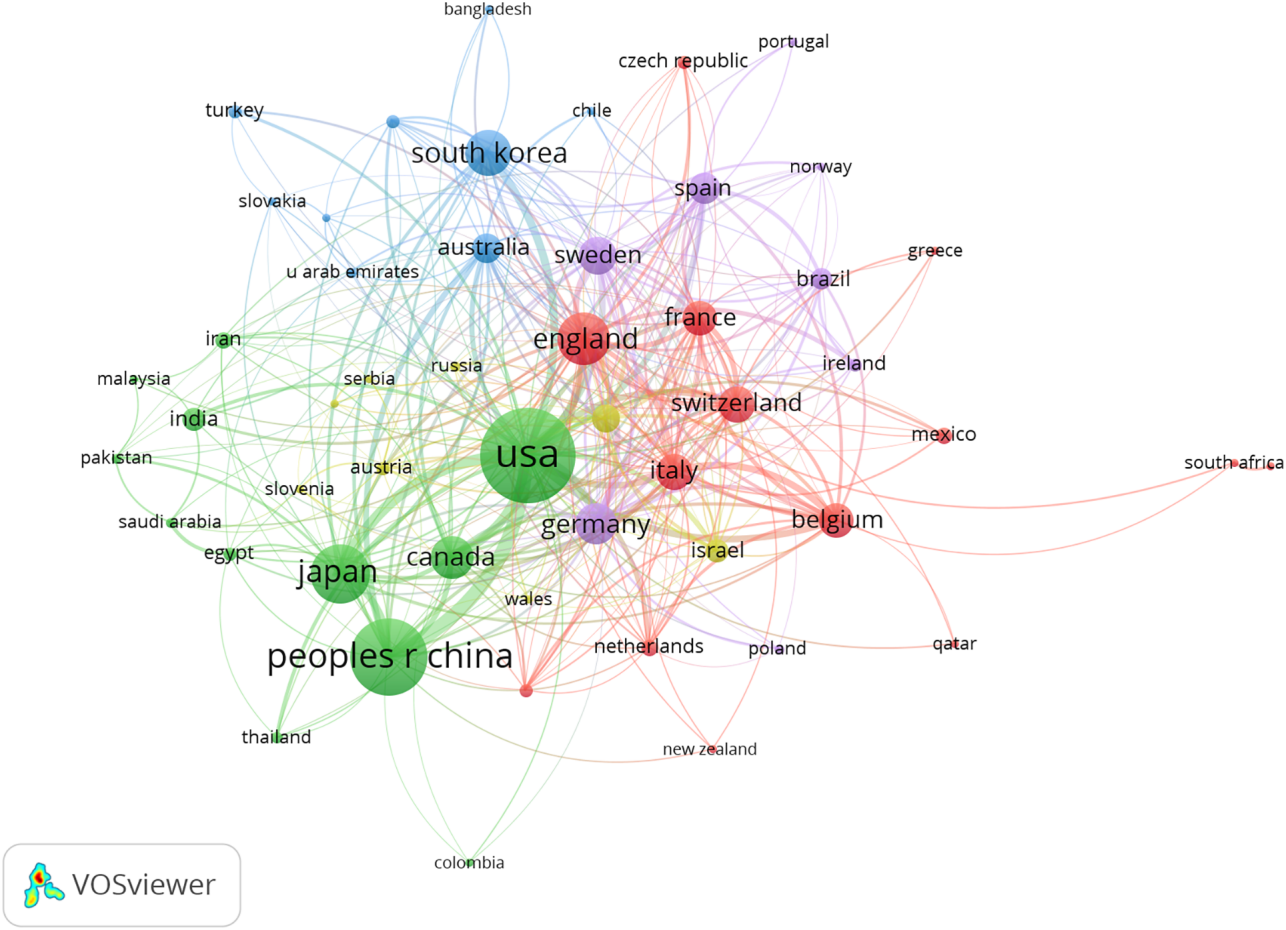
The co-occurrence map of countries/regions in beta-cell research. The total link strength was 1307; the layout parameters: Attraction: 2, Repulsion: -1. The circle size means the number of publications; the thickness of the line means the strength of the connection.

**Figure 4:**
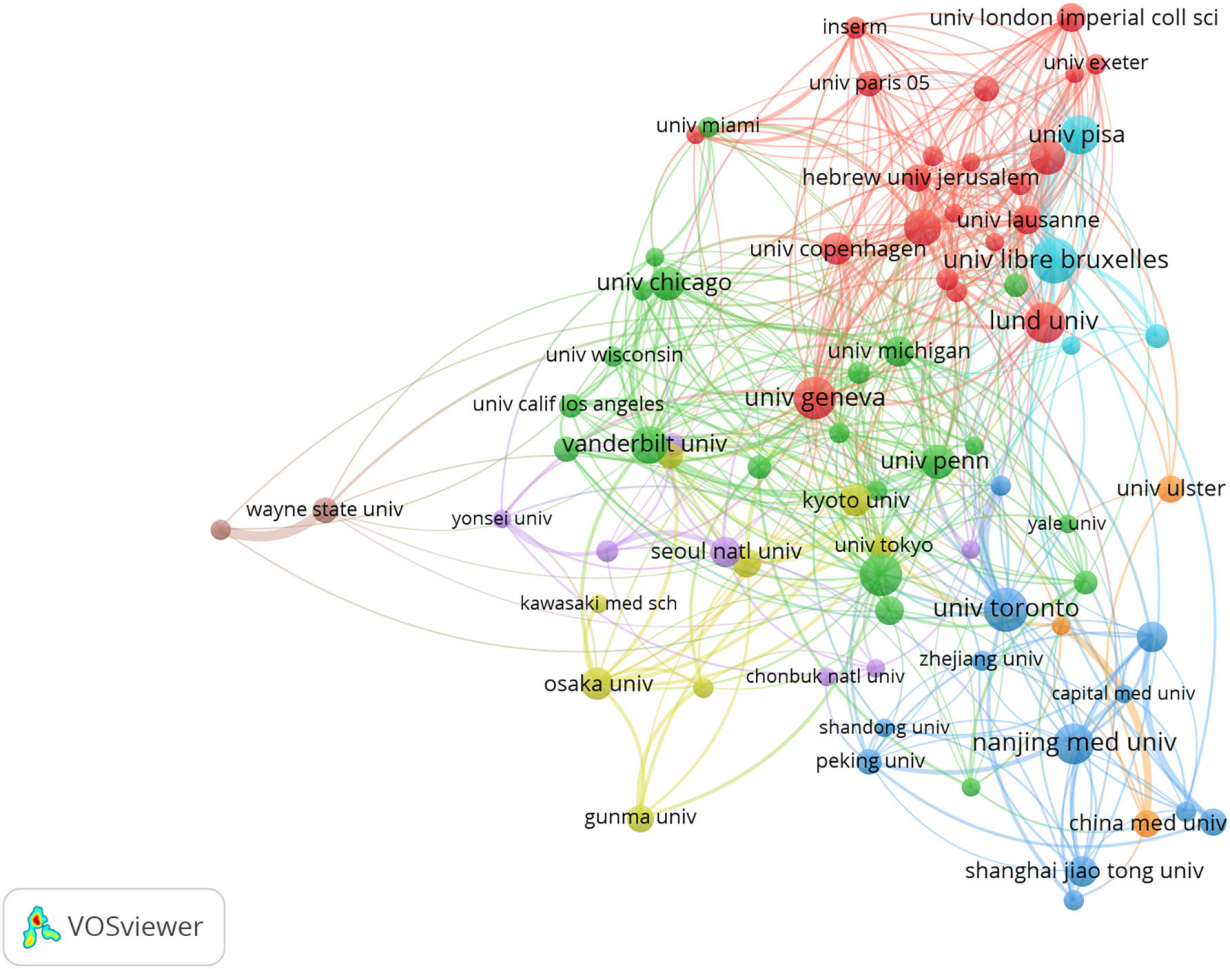
The co-occurrence map of institutions in beta-cell research. The total link strength was 633.50; the layout parameters: Attraction: 3, Repulsion: 1. Advanced parameters: Step size reduction: 1. The circle size means the number of publications; the thickness of the line means the strength of the connection.

A total of 4098 papers were published from 83 countries and 2938 institutions. As shown in **Table 1**, the largest number of publications originated from the United States (1197, 29,21%), followed by China (740, 19.33%), and Japan (472, 11.52%). **Figure 3** shows that the three countries had positive cooperation. The top 10 institutions published 721 articles in total. Universite libre de Bruxelles ranked first with 89 articles, followed by University of Toronto and University of Geneva. As shown in **Figure 4**, the top 3 universities had very little cooperation.

**Table 1:** Top 10 countries/regions and institutions related to beta-cell research.

Rank	country/region	N (%)	institution	N (%)
1	USA	1197 (29.21%)	univ libre bruxelles (Belgium)	89 (2.17%)
2	China	792 (19.33%)	univ Toronto (Canada)	82 (2.00%)
3	Japan	472 (11.52%)	univ Geneva (Switzerland)	77 (1.88%)
4	England	366 (8.93%)	Harvard univ (USA)	76 (1.85%)
5	South Korea	286 (6.98%)	Nanjing med univ (China)	74 (1.81%)
6	Canada	242 (5.91%)	Lund univ (Sweden)	72 (1.76%)
7	Germany	228 (5.56%)	univ Pisa (Italy)	67 (1.63%)
8	Sweden	197 (4.81%)	Vanderbilt univ (USA)	64 (1.56%)
9	Switzerland	182 (4.44%)	karolinska inst (Sweden)	62 (1.51%)
10	Italy	174 (4.25%)	univ Oxford (England)	58 (1.42%)

### 3.3 Authors and co-cited authors

**Figure 5:**
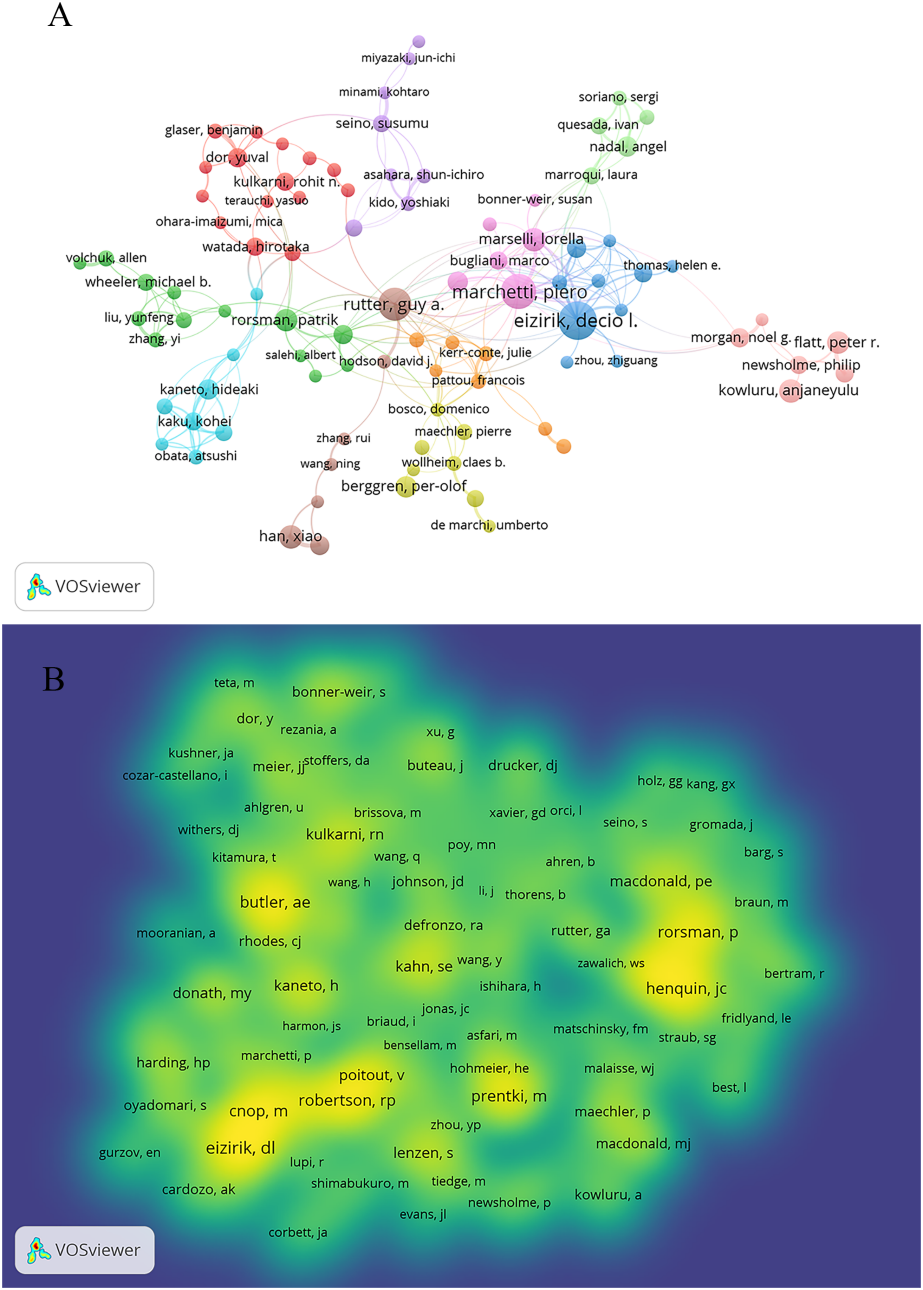
Authors and co-cited authors in beta-cell research. **(A)** The co-authorship network analysis of authors. The layout parameters: Attraction: 2, Repulsion: -2. The circle size means the number of publications; the thickness of the line means the strength of the connection; the circle colors mean different clusters; **(B)** The density map of co-cited authors. The layout parameters: Attraction: 2, Repulsion: 1. The color brightness means the frequency of occurrence.

A total of 19185 authors were involved in the publication of literature on beta-cell research. Among them, 106 authors published at least ten papers (**Figure 5A**). Decio I Eizirik published the most papers (n = 62), followed by Piero Marchetti (n = 53), and Guy A Rutter (n = 48) (**Table 2**). There were eleven colors in **Figure 5A**, representing 11 clusters among authors. Active collaborations usually exist in the same cluster, such as Piero Marchetti, Marco Bugliani, Susan Bonner-Weir, and Lorella Marselli. There were collaborations among linked nodes in different clusters, such as Decio L Eizirik, Piero Marchetti, and Guy A Rutter.

**Table 2:** Top 10 authors and co-cited authors related to beta-cell research.

Rank	Author	Count	Co-cited author	Co-citation
1	Decio L Eizirik	62	Decio L Eizirik	676
2	Piero Marchetti	53	Alexandra E. Butler	561
3	Guy A Rutter	48	Miriam Cnop	536
4	Lorella Marselli	29	J C Henquin	530
5	Xiao Han	27	R Paul Robertson	520
6	Anjaneyulu Kowluru	27	Marc Prentki	500
7	Peter R Flatt	25	Patrik Rorsman	489
8	Patrik Rorsman	25	Frances M Ashcroft	457
9	Per-Olof Berggren	24	Hideaki Kaneto	385
10	Hideaki Kaneto	22	Vincent Poitout	377

Co-cited authors are authors who have been co-cited together in a series of publications. Among 62007 co-cited authors, 119 were co-cited over 100 (**Figure 5B**). **Figure 5B** presents them as a density map, which could clearly show the high-frequency co-cited authors. The more citations, the brighter the color. As shown in **Table 2** and **Figure 5B**, Decio L Eizirik, Alexandra E. Butler, and Miriam Cnop had the most co-citations.

### 3.4 Journals and co-cited journals

A total of 4098 papers were published in 810 academic journals. The top 10 journals (**Table 3**) published 1191 papers, accounting for 29.06% of all publications. *Diabetes* published the highest number of studies (240, 5.86%), followed by *Journal of Biological Chemistry* (162, 3.95%), *Plos One* (147, 3.59%), and *Diabetologia* (142, 3.47%). *Diabetologia* had the highest IF of 10.460.

**Table 3:** Top 10 journals related to beta-cell research.

Rank	Journal	Count (%)	IF^#^ (2021)	JCR (2021)	Country
1	Diabetes	240 (5.86%)	9.337	Q1	USA
2	Journal of Biological Chemistry	162 (3.95%)	5.486	Q2	USA
3	Plos One	147 (3.59%)	3.752	Q2	USA
4	Diabetologia	142 (3.47%)	10.460	Q1	Germany
5	Biochemical and Biophysical Research Communications	127 (3.10%)	3.322	Q3	USA
6	Endocrinology	101 (2.46%)	5.051	Q2	USA
7	American Journal of Physiology-Endocrinology and Metabolism	87 (2.12%)	5.960	Q1	USA
8	Scientific Reports	68 (1.66%)	4.996	Q2	England
9	Molecular and Cellular Endocrinology	63 (1.54%)	4.369	Q2/Q3	Netherlands
10	Journal of Endocrinology	54 (1.32%)	4.669	Q2	England

^#^IF, Impact Factor.

Among 7023 co-cited journals, 31 journals had citations over 1,000. As shown in **Table 4**, *Diabetes* was the most co-cited journal (20019), followed by *Journal of Biological Chemistry* (13079), and *Diabetologia* (8470). *Nature* had the highest IF (69.504) among the top 10 co-cited journals, followed by *cell* (66.805), *Science* (63.798), and *Cell Metabolism* (31.373). Eight of ten co-cited journals were in the Q1 district of JCR, and the remainder were in Q2.

**Table 4:** Top 10 co-cited journals related to beta-cell research.

Rank	Co-cited Journal	Citation	IF^#^ (2021)	JCR (2021)	Country
1	Diabetes	20019	9.337	Q1	USA
2	Journal of Biological Chemistry	13079	5.486	Q2	USA
3	Diabetologia	8470	10.460	Q1	Germany
4	Proceedings of the National Academy of Sciences of the United States of America	6649	12.779	Q1	USA
5	Endocrinology	5219	5.051	Q2	USA
6	Journal of Clinical Investigation	5094	19.486	Q1	USA
7	Nature	4704	69.504	Q1	England
8	Cell	3271	66.850	Q1	USA
9	Science	2987	63.798	Q1	USA
10	Cell Metabolism	2893	31.373	Q1	USA

^#^ IF, Impact Factor.

### 3.5 Co-cited references and references burst

**Figure 6:**
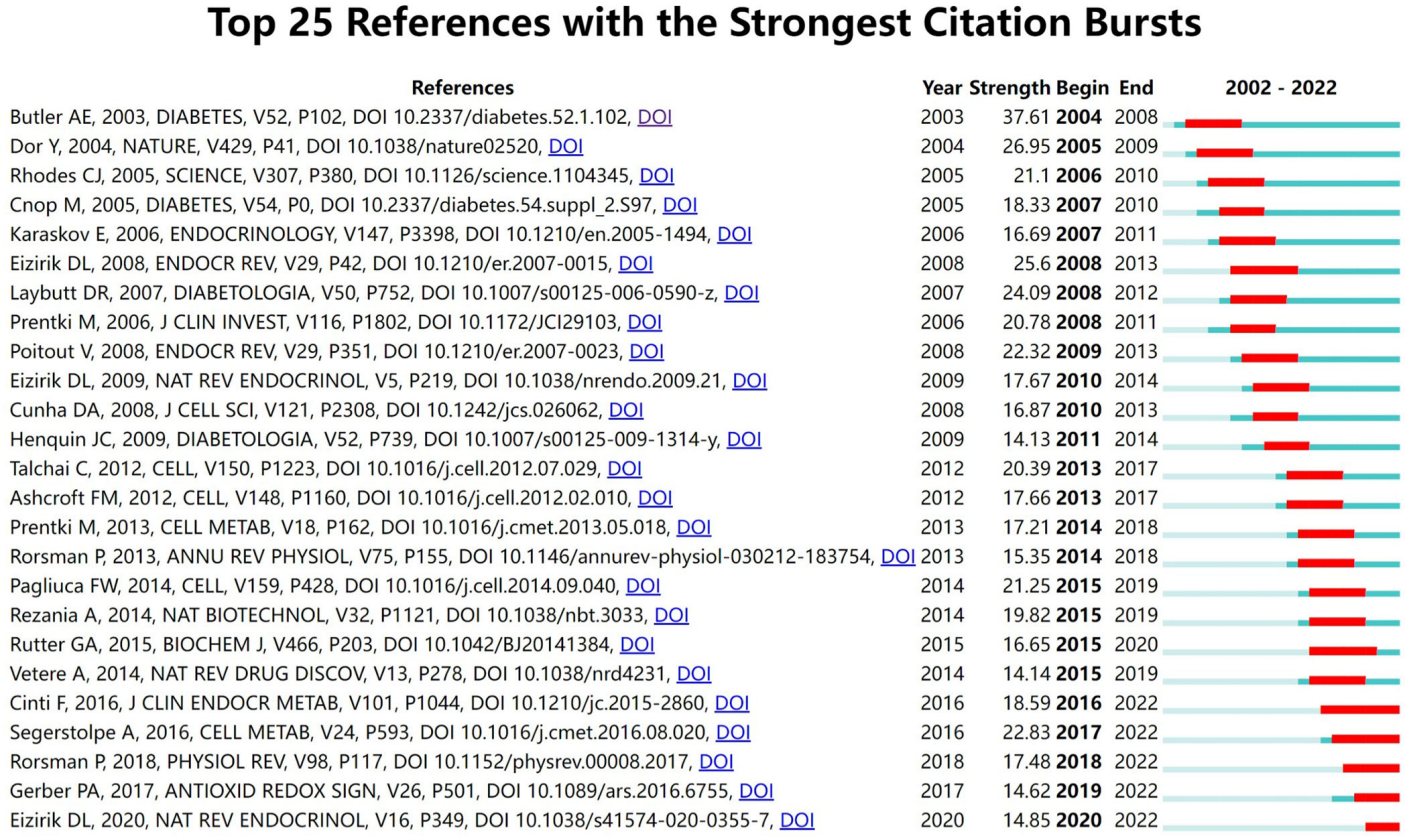
Top 25 references with the strongest citation bursts involved in beta-cell research.

CiteSpace was used to detect the co-cited references. Finally, 2047 co-cited references were retrieved, **Table 5** shows that the top 10 co-cited references were co-cited at least 45 times, of which “Beta-cell deficit and increased beta-cell apoptosis in humans with type 2 diabetes (9)” was the most frequently cited. **Figure 6** shows the top 25 references with the most robust citation bursts. And the earliest reference with citation bursts was in 2004. Notably, five references were still in burstness.

**Table 5:** Top 12 co-cited references related to beta-cell research.

Rank	ID	Title	Co-citation	Centrality
1	A. E. Butler (2003)	Beta-cell deficit and increased beta-cell apoptosis in humans with type 2 diabetes	91	0.01
2	Y Dor, (2004)	Adult pancreatic beta-cells are formed by self-duplication rather than stem-cell differentiation	73	0.08
3	D. L. Eizirik (2008)	The role for endoplasmic reticulum stress in diabetes mellitus	70	0.05
4	D. R. Laybutt (2007)	Endoplasmic reticulum stress contributes to beta cell apoptosis in type 2 diabetes	66	0.06
5	C. J. Rhodes (2005)	Type 2 diabetes-a matter of beta-cell life and death?	53	0.03
6	V. Poitout (2008)	Glucolipotoxicity: fuel excess and beta-cell dysfunction	53	0.02
7	A. Segerstolpe (2016)	Single-Cell Transcriptome Profiling of Human Pancreatic Islets in Health and Type 2 Diabetes	50	0.04
8	F. Cinti (2016)	Evidence of beta-Cell Dedifferentiation in Human Type 2 Diabetes	46	0.06
9	M. Cnop (2005)	Mechanisms of pancreatic beta-cell death in type 1 and type 2 diabetes: many differences, few similarities	45	0.21
9	M. Prentki (2006)	Islet beta-cell failure in type 2 diabetes	45	0.05
9	C. Talchai (2012)	Pancreatic beta cell dedifferentiation as a mechanism of diabetic beta-cell failure	45	0.03
9	F. W. Pagliuca (2014)	Generation of functional human pancreatic beta cells *in vitro*	45	0.02

### 3.6 The analysis of hotspots and frontiers

**Figure 7:**
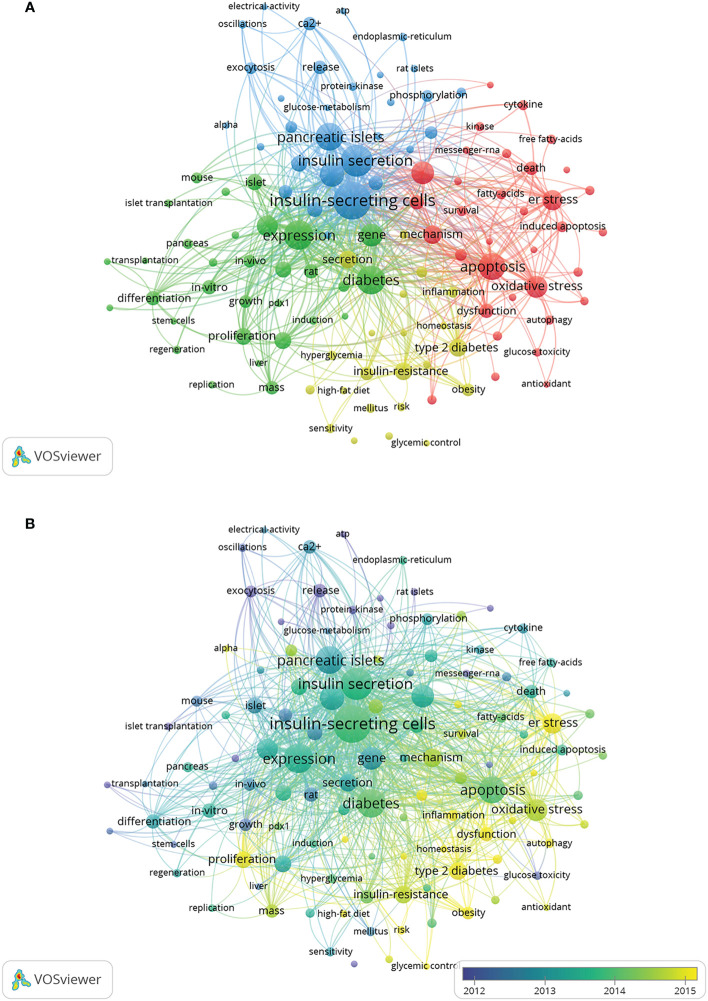
Keywords related to beta-cell research. **(A)** Network visualization of keywords drawn by VOSviewer. The layout parameters: Attraction: 2, Repulsion: 0. The circle size means the frequency of occurrence; the circle colors mean different clusters; **(B)** Overlay visualization of keywords drawn by VOSviewer. The layout parameters: Attraction: 2, Repulsion: 0. The circle size means the frequency of occurrence; the circle colors mean the average published year.

**Figure 8:**
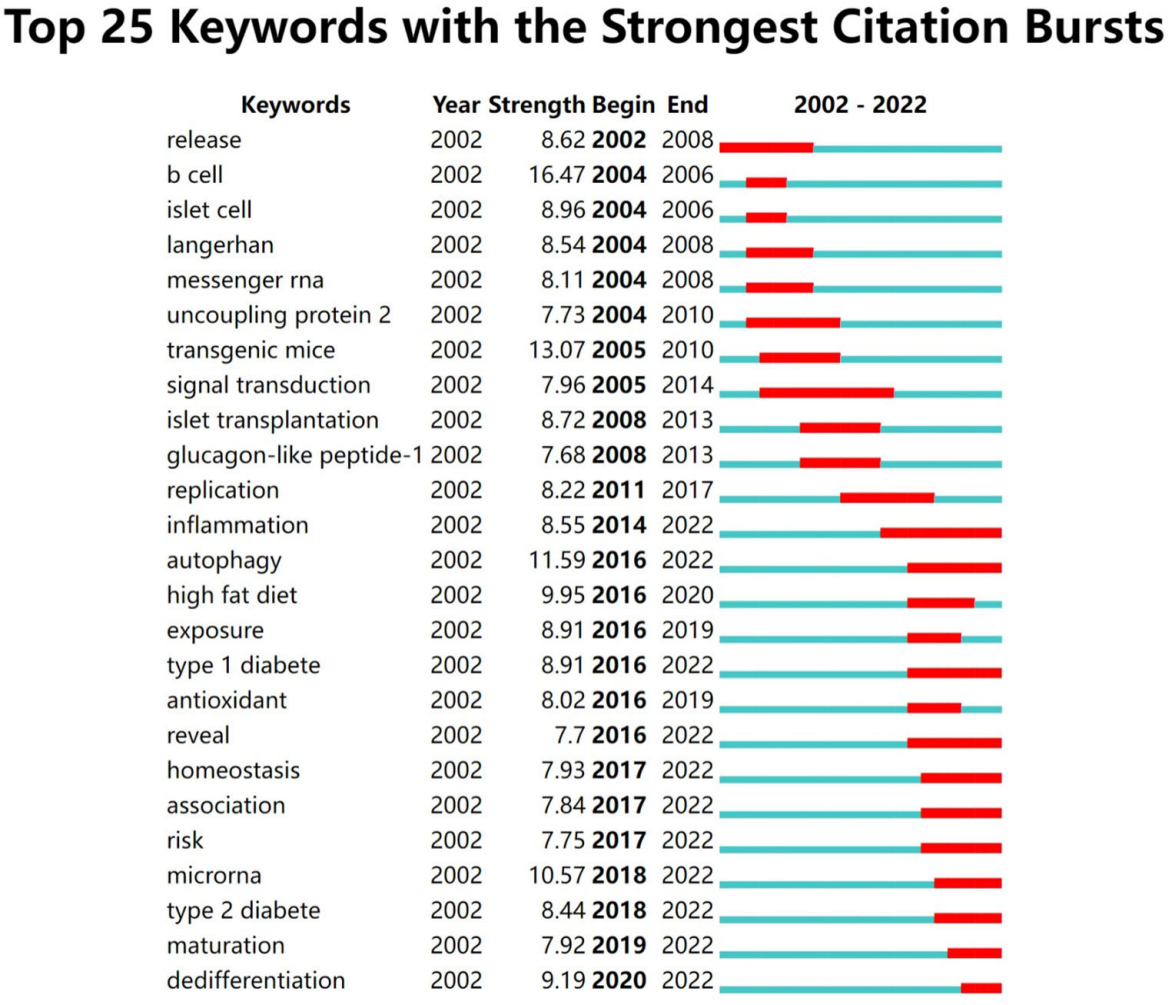
Top 25 keywords with the strongest citation bursts involved in beta-cell research.

A total of 11,774 keywords were extracted, of which 634 appeared more than ten times and 51 appeared more than 100 times. **Table 6** shows the top 20 keyword co-occurrence terms. “diabetes” (861), “apoptosis” (740), “oxidative stress” (494), “ER stress” (432), “insulin-resistance” (313), “proliferation” (293), and “transcription factor” (263) were core contents of beta-cell research. Cluster analysis can show the knowledge structure of the research field (10). As shown in **Figure 7A**, we can see the clusters of red, yellow, green, and blue, which respectively represent four different research directions. The main keywords of the red cluster are apoptosis, oxidative stress, ER stress, autophagy, and death, which are related to the pathological mechanism of beta-cell failure. The keywords of the yellow cluster focus on the risk factor related to beta cells, which include insulin-resistance, obesity, hyperglycemia, and adipose tissues. While the keywords of the green cluster are related to the recovery of beta cells, including proliferation, growth, regeneration, replication, mass, stem cells, transplantation, and pdx1. The blue cluster mainly includes insulin-secreting cells, glucose-metabolism, exocytosis, and electrical-activity, which are related to the physiology of beta cells. **Figure 8** shows the top 25 keywords with the most robust citation bursts. Notably, inflammation, autophagy, type 1 diabetes, risk, microRNA, type 2 diabetes, maturation, and dedifferentiation were in burstness until 2022. The overlay map (**Figure 7B**) shows that ER stress, inflammation, autophagy, proliferation, obesity, homeostasis, risk, and glycemic control are emerging fields that were colored yellow.

**Table 6:** Top 20 keywords of beta-cell research.

Rank	Keywords	Counts	Rank	Keywords	Counts
1	insulin-secreting cells	1463	11	insulin	447
2	insulin secretion	1054	12	ER stress	432
3	expression	870	13	mechanism	354
4	diabetes	861	14	type 2 diabetes	323
5	pancreatic islets	808	15	secretion	317
6	apoptosis	740	16	insulin-resistance	313
7	glucose	635	17	proliferation	293
8	gene	526	18	mice	292
9	activation	518	19	transcription factor	263
10	oxidative stress	494	20	islet	262

## 4 Discussion

### 4.1 General information

The number of papers published in each period reflects the development trend of research in this field. From 2002 to 2003, only 3 articles were published, which meant that the study of beta cells had not received much attention. Notably, Alexandra E. Butler et al. published the most co-cited article with the strongest citation bursts, where they found that beta-cell apoptosis was the underlying mechanism of beta-cell failure in 2003 (9). This was a breakthrough in the field of beta cell failure. Since then, a number of researchers had begun focusing on beta cells and published related articles with a steady upward trend.

According to **Table 1** and **Figure 3**, the United States made the largest contribution to beta-cell research, accounting for nearly 30% of the published papers, and cooperated closely with many countries around the world, such as China, Japan, South Korea, Canada, England, Germany, etc. As shown in **Figure 4**, most of the cooperative institutions have closer domestic cooperation, but relatively less international cooperation.

Among the top 10 authors and co-cited authors, Decio L Eizirik not only published the most papers but also had the most co-citations, indicating his outstanding contribution to beta-cell research. Eizirik is a professor at Univ Libre Bruxelles, focusing on mechanisms related to diabetes and pancreatic islet cells, such as ER stress, inflammation, apoptosis, etc. In 2008, his group published a review that described the mechanisms of ER stress in diabetes and outlined some of the areas for future beta-cell research, which may contribute to clarifying the role of ER stress in diabetes. It was co-cited up to 70 times and had a strong citation burst. Meanwhile, we found two other scholars, namely Patrik Rorsman and Hideaki Kaneto, who were not only the top 10 productive authors but also the top 10 co-cited authors, also had an outstanding contribution to beta-cell field. Rorsman is an electrophysiologist from The Oxford Centre for Diabetes, Endocrinology, and Metabolism, focusing on the electrophysiology of beta-cell (1). He published a review in Physiological reviews in 2018, detailing the electrophysiology of beta cells and insulin secretion and offering new ideas for intervening in ion channels to treat diabetes. Hideaki Kaneto is a professor at Kawasaki Medical School, who focuses on the effects of relevant transcription factors (PDX1, MafA) and anti-diabetic drugs on beta-cell function (11–14).

Journals and co-cited journals analysis (**Tables 3**, **4**) showed that *Diabetes* published the most insulin-secreting cells studies, and received the largest number of co-citations. *Journal of Biological Chemistry*, *Diabetologia*, and *Endocrinology* were the top 10 publication journals and top 10 co-cited journals, indicating their essential role in disseminating beta-cell research. The highest IF journals such as *Cell*, *Science*, *Cell Metabolism*, and *Nature* were the top ten co-cited journals. The papers published in them may provide a theoretical basis for future research.

### 4.2 Knowledge base

Co-cited references are two or more references that have been cited together by other publications (4). And the knowledge base is the collection of co-cited references cited by the corresponding research community, which is not entirely equivalent to highly cited references. In this study, we used CiteSpace to analyze co-cited references, of which the top 12 were listed below.

In 2003, *Diabetes* published the most co-cited study co-authored by Alexandra E. Butler and other five scholars (9). This study examined pancreatic tissue from 124 autopsies and measured relative beta-cell volume, frequency of beta-cell apoptosis and replication, and new islet formation from exocrine ducts. And it finally demonstrated that beta-cell mass was decreased in type 2 diabetes and the underlying mechanism was increased beta-cell apoptosis rather than decreased beta-cell replication. Alexandra E Butler et al. suggested that therapeutic approaches designed to arrest apoptosis could be a significant development in the management of type 2 diabetes, and a new approach may reverse the disease to some extent, rather than just palliate glycemia.

Yuval Dor et al. (15) published the second co-cited study in *Nature* in 2004. A method for genetic lineage tracing was used to determine the contribution of stem cells to pancreatic beta cells. The authors found that pre-existing beta cells, rather than pluripotent stem cells, are the major source of new beta cells during adult life and after pancreatectomy in mice. They demonstrated that terminally differentiated beta cells retain significant proliferative capacity *in vivo* and questioned the notion that adult stem cells had a significant role in beta cell recruitment.

The third co-cited paper was published by Decio L Eizirik et al. in *Endocrine Reviews* in 2008 (16). This review summarized the role of ER stress in diabetes and illustrated the transition between “normal” and “pathological” ER stress. Meanwhile, they discussed the molecular mechanism of ER stress in beta-cell apoptosis and beta-cell death, and the recovery of beta cells from ER stress. Additionally, the link between ER stress and insulin resistance, and obesity was presented.

*Diabetologia* published the fourth co-cited experiment research by D. R. Laybutt et al. in 2007 (17). This study used insulin-secreting MIN6 cells exposed to elevated lipids, islets isolated from db/db mice and pancreas sections of humans with type 2 diabetes to evaluate the expression of genes involved in ER stress and demonstrated ER stress occurred in type 2 diabetes and was required for aspects of the underlying beta-cell failure.

In 2008, Vincent Poitout et al. (18) published the fifth co-cited review article in *Endocrine Review*. They presented evidence supporting the concept of glucotoxicity, and described the underlying mechanisms with an emphasis on the role of oxidative stress. In addition, they discussed the functional manifestations of glucolipotoxicity on insulin secretion, insulin gene expression, and beta-cell death.

Christopher J. R hodes (19) published the sixth co-cited article in *science* in 2005. He thought the failure that beta-cell mass failed to compensate for insulin resistance was caused by a significant increase in beta-cell apoptosis, and highlighted the role of IRS-2 signaling in beta-cell survival.

In 2016, *Cell metabolism* published the seventh co-cited paper by Asa Segerstolpe and 13 other scholars (20). In this study, they used single-cell transcriptomics to generate transcriptional profiles of individual pancreatic endocrine and exocrine cells of healthy and type 2 diabetic donors and revealed cell-type-specific gene expression and novel subpopulations, as well as gene correlations to BMI and gene expression alterations in diabetes.

The eighth co-cited paper was published by Francesca Cinti, et al. in *Journal of Clinical Endocrinology and Metabolism* in 2016 (21). This study surveyed pancreatic islets from 15 diabetic and 15 nondiabetic organ donors and proved that beta cells become dedifferentiated and transdifferentiated to α- and σ- “like” cells in human type 2 diabetes.

In 2017, *Diabetes* published the ninth co-cited study authored by Miriam Cnop et al. (22). This review summarized the similarities and differences between the mechanisms of beta-cell death in type 1 diabetes and type 2 diabetes.

In 2014, the tenth co-cited paper was published by Felicia W. Pagliuca et al. in *Cell* (23). They successfully generated hundreds of millions of glucose-responsive beta cells from human pluripotent stem cells *in vitro*. These cells can mimic the function of human islets *in vitro* and *in vivo* and secrete human insulin into the serum of mice shortly after transplantation in a glucose-regulated manner, ameliorating hyperglycemia in diabetic mice.

Chutima Talchai et al. (24) published the eleventh co-cited article in *Cell* in 2012. This study used mice lacking foxo1 in beta-cells to elucidate the contribution of beta-cell number or function to beta-cell dysfunction and found that dedifferentiation trumps endocrine cell death in the natural history of beta-cell failure. Furthermore, this study emphasized a foxo1-dependent mechanism that prevents dedifferentiation and suggested that the treatment of beta-cell dysfunction should recover differentiation, rather than promote beta-cell replication.

*Journal of Clinical Investigation* published the twelfth review by Marc Prentki et al. in 2006 (25). This review summarized the mechanisms of beta-cell failure in the pathogenesis of obesity-related type 2 diabetes and the mechanisms involved in the compensation process for insulin resistance.

Generally, the top 12 co-cited references focused on reviews (three reviews were published in 2006 and 2008), and mechanisms (including apoptosis, dedifferentiation, trans-differentiation, ER stress, regeneration, etc.), all of these were the research foundations of beta cells.

### 4.3 Hotspots evolution and emerging topics

In bibliometrics, keyword co-occurrence can reflect the hotspot of an academic field (26), the overlay map can present the evolution of new hotspots, and keyword burst and reference citation bursts can characterize the emerging topics in a discipline (27). In this study, we attempted objectively to evaluate the hotspots and frontiers of research on insulin-secreting cells by analyzing keyword co-occurrence (**Figure 7A**), keyword overlap (**Figure 7B**), keyword burst (**Figure 8**), and reference burst (**Figure 6**).

Keyword analysis showed that diabetes was closely related to beta cells and that how to protect and restore beta cells was the focus of research. Apoptosis, oxidative stress, and ER stress were the most studied mechanisms of beta-cell failure in the past 20 years. As research goes on, emerging topics occurred continuously. In the last decade, miRNA, autophagy, dedifferentiation, and inflammation have emerged as novel mechanisms for the study of beta-cell failure, while the risk factors associated with beta cells, such as obesity and insulin resistance are also the hotspots and emerging research trends.

References with strong citation bursts could also feature the emerging topics of a field (4). Among the top 25 references with the strongest citation bursts, five studies are still in the bursting phase today. These articles represent the latest emerging topics of beta-cell research, and indicate future potential research directions, such as beta-cell dedifferentiation, single-cell transcriptome, oxidative stress, hypoxia, beta-cell electrical activity, and gene expression (1, 20, 21, 28, 29). Among the other 20 references, five studies mainly elaborated on the physiology of beta cells regulating of insulin secretion in human pancreatic islets (3, 15, 30–32); eight studies focused on the pathological mechanisms of beta-cell failure, mainly including apoptosis, dedifferentiation, ER stress, inflammation (9, 16, 17, 19, 22, 24, 33, 34); three studies were related to the treatment and reversal of diabetes targeting the beta cells (23, 35, 36); four studies were primarily associated with risk factors of beta-cell failure (18, 25, 37, 38), such as obesity, glucolipotoxicity, and free fatty acid. As we can see, the results of reference burst analysis are basically consistent with the keyword analysis.

According to the above analysis, we summarized three aspects as follows.

#### 4.3.1 Beta-cell electrical activity and insulin secretion

Beta-cell electrical activity and insulin secretion are important parts of beta-cell physiology. Insulin secretion is closely associated with beta-cell electrical activity. Beta-cell has electrical excitability like nerve cells, which is hyperpolarized (−70 mV) and electrically silent at low glucose (1). Notably, glucose induces a concentration-dependent depolarization. As the glucose concentration increases, beta-cell becomes gradually more depolarized, and once the membrane potential exceeds −60 mV, electrical activity and insulin secretion are initiated (39, 40). It is currently known that insulin is released *via* exocytosis regulated with the related ion channels. Among them, the ATP-sensitive K^+^ channel (K_ATP_ channel) is the major ion channel open at rest in beta-cells, which is of critical significance for insulin secretion. When the K_ATP_ channel is closed with glucose or sulfonylureas (41), this depolarizes the beta-cell and initiates beta-cell electrical activity, Ca^2+^influx, and insulin secretion. It was found that most patients with neonatal diabetes caused by K_ATP_ channel gene mutations retain sufficient beta-cells to control glycemia when the open K_ATP_ channels are closed with sulfonylureas (42). Perhaps, this provides a novel idea that we just need to wake up the resting beta-cells in the treatment of type 2 diabetes.

#### 4.3.2 Pathogenesis of beta-cell failure

Over the past two decades, the pathogenesis of beta-cell failure can be broadly divided into two stages. Before 2012, apoptosis is an important mechanism of beta-cell failure. Alexandra E. Butler et al. (9) laid the foundation for the study of apoptosis in beta-cell failure by demonstrating that apoptosis is the main cause of beta-cell mass reduction through examining pancreatic tissue from 124 autopsies in 2003. ER stress, mitochondrial dysfunction, and oxidative stress play a central role in promoting beta cell dysfunction and they are closely interrelated and aggravate each other (43).

After 2012, however, dedifferentiation became another important mechanism of beta-cell failure. In 2012, Chutima Talchai et al. identified beta-cell dedifferentiation as the mechanism of beta-cell failure and suggested that beta-cell dedifferentiation is more important than apoptosis (24). Since then, increasing evidence has demonstrated that beta-cell dedifferentiation is the major cause of the loss of beta-cell mass and function (21, 44, 45). Further, oxidative stress, inflammation, ER stress, miRNAs, and lncRNAs are related to the mechanisms of beta-cell dedifferentiation (46–49). Interestingly, although Alexandra E. Butler (9) and Chutima Talchai (24) did not share the same understanding of beta-cell failure, they both suggested that treatment of beta-cell failure should not promote cellular proliferation or replication and hoped to reverse diabetes, rather than just control glycemia.

Both apoptosis and dedifferentiation are associated with oxidative stress and ER stress. Oxidative stress is the result of an imbalance between the cellular antioxidant defense system and the production of reactive oxygen species (47). The oversupply of nutrients, including glucose and fatty acids, can lead to the production of reactive oxygen species (28, 50). Unfortunately, beta cells have poor endogenous antioxidant capacity (51), making beta cells highly susceptible to oxidative stress. Antioxidant therapy is a hotspot for beta-cell failure. Many studies have shown that several antioxidant supplements increased the proliferation rate of beta cells, suggesting antioxidants as a new treatment for diabetes (52). ER stress results from the accumulation of unfolded or misfolded proteins in the ER space (53). The endoplasmic reticulum is a major site of proinsulin folding and the main intracellular Ca^2+^ reservoir (54), therefore, its homeostasis is critical for insulin secretion as well as beta-cell function and survival (55). It was demonstrated that ER stress occurs in type 2 diabetes and is required for various aspects of underlying beta-cell failure (17). Notably, the accumulation of unfolded proteins in the ER leads to oxidative stress and subsequent damage associated with beta-cell dedifferentiation or apoptosis (56, 57).

#### 4.3.3 Stem cells for beta-cell replacement

Current drug treatments cannot completely prevent or reverse the progression of beta-cell failure. Fortunately, islet transplantation is a promising alternative to beta-cells in the future (58). Over the past 20 years, researchers have made great progress in beta-cell replacement. In the early 2000s, there was insufficient evidence that stem cell-derived cells could respond to glucose. Until 2014, glucose-responsive pancreatic beta-cells can be cultured *in vitro* from human pluripotent stem cells (PSCs) and alleviate hyperglycemia in diabetic mice (23, 35). Remarkably, there was substantial evidence about Phase I and Phase II clinical trials that provided a possibility of translating the theory of PSCs-derived pancreatic islets into clinical application (59, 60). However, Stem cell replacement therapies still face some problems, including the assessment of beta cell function, the protection of beta cells after transplantation, immune rejection, and so on (61, 62), and overcoming these problems might become the focus of future research.

From the above analysis, we can summarize the evolution of key mechanisms and therapeutic approaches to beta-cell failure. In earlier studies, apoptosis was a hotspot for research on beta-cell failure, while in the last decade, beta-cell dedifferentiation has become a new trend in research. Oxidative stress and ER stress have been hot spots in the study of beta-cell failure. In addition, inflammation, autophagy, miRNA, and lncRNA are also hot topics nowadays. Additionally, stem cell replacement therapies might be the alternative way to reverse diabetes. Restoring beta-cell mass and function will remain a research goal in the future.

## 5 Limitations

Data were obtained from the WoSCC database; therefore, studies not collected in the WoSCC were omitted. However, WoSCC is the most widely used database for scientometric analysis and includes most information in relevant articles (5). In addition, WoSCC has more accurate document-type assignments than Scopus (63).The uneven quality of data might undermine the credibility of the knowledge mapping, as reported by other bibliometric studies (64). Nevertheless, the visualization-based literature analysis lays the foundation for researchers to understand hotspots and potential problems in beta-cell research.

## 6 Conclusion

In conclusion, beta-cell research is in a steadily developing phase with active cooperation around the world, of which the United States is the major collaborating center. Decio L Eizirik contributed to most of the publications, and had the most co-citationsin beta-cell research. Current beta-cell studies are focused on beta-cell electrophysiology, pathogenesis and risk factors of beta-cell failure, and beta-cell replacement therapy. Notably, apoptosis and dedifferentiation have still been vital mechanisms of beta-cell failure until now. Further, ER stress and oxidative stress are core mechanisms of beta-cell apoptosis and dedifferentiation. Among them, inflammation, autophagy, miRNA, and lncRNA might be the rising and promising research areas about the mechanisms of beta-cell failure.

Overall, this is the first study to systematically analyze beta-cell-related publications by bibliometric and knowledge-map. Compared to traditional reviews, this study provided an objective and comprehensive overview of beta-cell research through bibliometric and visual methods. The information would provide helpful references for scholars focusing on beta cells.

## Data availability statement

The raw data supporting the conclusions of this article will be made available by the authors, without undue reservation.

## Author contributions

GZ and ZC designed this study, YL collected the data and performed the analysis, YL and TW normalized the pictures, YL and TW wrote the original draft. All authors contributed to the article and approved the submitted version.
